# Implementation of Deep-Learning-Based CSI Feedback Reporting on 5G NR-Compliant Link-Level Simulator †

**DOI:** 10.3390/s23020910

**Published:** 2023-01-12

**Authors:** Daniel Gaetano Riviello, Riccardo Tuninato, Elisa Zimaglia, Roberto Fantini, Roberto Garello

**Affiliations:** 1 Department of Electrical, Electronic, and Information Engineering, University of Bologna, 40136 Bologna, Italy; 2 Department of Electronics and Telecommunications (DET), Politecnico di Torino, 10129 Torino, Italy; 3 TIM S.p.A., 10148 Torino, Italy

**Keywords:** 5G, New Radio, deep learning, convolutional neural network, CSI reporting

## Abstract

Advances in machine learning have widened the range of its applications in many fields. In particular, deep learning has attracted much interest for its ability to provide solutions where the derivation of a rigorous mathematical model of the problem is troublesome. Our interest was drawn to the application of deep learning for channel state information feedback reporting, a crucial problem in frequency division duplexing (FDD) 5G networks, where knowledge of the channel characteristics is fundamental to exploiting the full potential of multiple-input multiple-output (MIMO) systems. We designed a framework adopting a 5G New Radio convolutional neural network, called NR-CsiNet, with the aim of compressing the channel matrix experienced by the user at the receiver side and then reconstructing it at the transmitter side. In contrast to similar solutions, our framework is based on a 5G New Radio fully compliant simulator, thus implementing a channel generator based on the latest 3GPP 3-D channel model. Moreover, realistic 5G scenarios are considered by including multi-receiving antenna schemes and noisy downlink channel estimation. Simulations were carried out to analyze and compare the performance with current feedback reporting schemes, showing promising results for this approach from the point of view of the block error rate and throughput of the 5G data channel.

## 1. Introduction

New Radio (NR) wireless networks are anticipated to support very high data rates and new applications that will create opportunities for a radically new radio technology paradigm [1]. In this context, machine learning (ML) has regained attention for its achievements especially in the upper layers, such as in cognitive radio [2,3], self-organizing networks (SONs) [4] and resource management [5,6]. However, the authors of [7] claim that the potential use of deep learning (DL) to the physical layer will be increasingly recognized. This appears to be reasonable considering the new features of next-generation communications, such as complex scenarios, with a consequent lack of information about the channel model, high data rates and accurate processing requirements [7].

Studies on ML applications to the physical layer have been carried out, for example, for modulation recognition [8,9], signal classification [10], multiple input multiple output (MIMO) detection [11], channel state information (CSI) feedback reduction [12,13], non-orthogonal multiple access (NOMA) [14], hybrid precoding [11,15], encoding and decoding [16], and channel estimation and equalization [17,18,19,20]. Researchers have presented a range of arguments to support the adoption of DL techniques in contemporary communication environments [7,11,16]:

- deep networks are very good and adaptable function approximators—they learn the weights of the model optimizing the overall performance through a training process, instead of requiring a rigorous mathematical description that could be hard to extract in complex scenarios;
- DL-based systems could replace manual feature extractors and learning features automatically, flexibly adapting the structure and the parameters of the model, with considerable improvement to the end-to-end performance;
- DL-based algorithms work very well with large amounts of data due to their intrinsic parallel and distributed computing, leading to speeding up of computation;
- DL models can overcome the block structure to provide a complete view of the system and optimize it end-to-end.

This paper proposes a DL-based solution for CSI feedback improvement in 5G-based communication systems. The availability of information about the colorful characteristics of the communication channels has a fundamental impact on many transmission features in most modern radio-access technologies [21]. This framework of information can vary from a rough estimate of the radio link path loss, which is useful for adjusting the transmitted power, to very precise knowledge of the channel amplitude and phase [21]. Measurements and estimates can be performed by either the transmitter or the receiver side. Focusing on the downlink case, the knowledge of the channel characteristics can be acquired by means of device measurements or by the network itself, depending on the particular scenario:

- when working in frequency division duplexing (FDD) mode, downlink and uplink could potentially be significantly different. In consequence, to obtain reliable information about the downlink channel, measurements must necessarily be acquired by the user device and then reported to the network; the network will exploit this feedback to properly set the transmission parameters for future downlink transmissions;
- in time-division duplexing (TDD) communications, when downlink and uplink transmissions experience the same channel characteristics, feedback from the device is not necessary, since the network itself can obtain useful information about the downlink features of interest by measuring them in the uplink transmissions.

In general, the evaluation of the radio environment is referred to as channel sounding and requires the adoption of specific reference signals (RS) on which the receiver can perform measurements.

The adoption of appropriate channel modelling is critical when performing simulations involving signal processing, such as channel sensing. Several channel models have been reported in the literature, with two main approaches described: stochastic and statistical. The former relies on the definition of the probability density function (PDF) of the channel parameters depending on the geometry of the environment, while the latter exploits real measures on which to base the definition of the channel. Each of these solutions has possible drawbacks, but a hybrid approach can be chosen, firstly, stochastically, characterizing the channel parameters through their PDF, and subsequently leveraging real data to confirm and eventually adapt them. In this investigation, we rely on the latest 3D stochastic channel model for 5G mmWave massive MIMO communications released by 3GPP in TR 38.901 [22,23]. It covers the entire range of 0.5–100 GHz, with particular relevance to the new challenges represented by mmWave communications, where some phenomena, such as path attenuation, noise, interference, and atmospheric attenuation, can seriously impact the signal transmission. One of its main characteristics is the modelling of the channel through a certain number of clusters distributed over the area, which groups the scatterers and so produces different replicas of the propagated signal (i.e., different rays). It is categorised as a clustered delay line (CDL) model. Moreover, while, in 4G, the propagation waves were considered only in the horizontal plane, thus producing a 2D channel model, mmWave communications (and thus 5G) require a 3D channel model, characterising the signal propagation depending on the elevation coordinate. The channel model generation is performed inside a link-level simulator designed by TIM, which also provides the resource blocks generation, transmission and reception for a 5G 3GPP-compliant communication, with evaluation of the primary downlink shared channel (PDSCH) throughput and the block error rate (BER).

### 1.1. Literature Review

Several studies, such as [12,13,24,25], suggest that, with the introduction of massive MIMO technologies in 5G systems, the potential gain from an accurate estimation of channel characteristics will considerably increase, with channel sounding becoming a critical issue. Moreover, as the authors of [12,13] point out, the most-used approaches to reduce the feedback overhead, based on codebooks or vector quantization, process feedback quantities whose dimensions scale with the number of antennas, making these techniques unsuitable in massive MIMO scenarios. These criticisms have stimulated new studies aimed at overcoming the difficulties associated with traditional approaches with DL-based solutions.

The authors of [12] designed an autoencoder based on neural networks to compress and then reconstruct the channel state information, called CsiNet. This paper stimulated significant interest among researchers who sought to expand the applicability and improve the performance. In [26], the authors present two different structures, ConvCsiNet and ShuffleCsiNet, both able to outperform CsiNet from an NMSE point of view. Even though the latter has reduced complexity with respect to the first structure, it is still more complex than CsiNet. One of the most recent advancements in DL is the concept of attention, which is exploited in [27,28]. In the former, long short-term memory (LSTM) is adopted to learn the temporal correlation of channels, while an attention mechanism is developed to perceive local information and automatically weight feature information. This kind of solution can further improve the reconstruction capabilities, but comes at a higher cost that could not be supported in terms of user equipment. A solution to effectively decrease the complexity of CsiNet was proposed in [29], which identified a lighter, fully connected (FC) layer as the key to encoder compression. The network parameters are transformed from a 32-bit floating point number to a 1-bit binary number, optimizing a positive scale factor that minimises the distance between standard weights and binary weights. In [30], a DL-based CSI feedback framework is described for beam-forming (BF) design, CsiBFnet. Its goal is to maximize the BF performance gain rather than the feedback accuracy, including with respect to single-cell or multi-cell scenarios. Another interesting solution is the CQNet described in [31], which focuses on the impact of quantisation on the channel estimation coefficients and exploits radial coordinates to improve quantisation scheme efficiency.

### 1.2. Paper Contribution and Organization

The solutions presented in the papers referred to above increase the possibilities of DL-based solutions for CSI; however, there is a lack of studies focusing on the characteristics of a system based on 5G NR and, therefore, the channel model and performance in terms of the BLER of the data channel. In this article, we extend our findings in [32] and propose a DL-based technique for channel state information feedback reporting in FDD transmission mode, based on the implementation of a convolutional neural network, called NR-CsiNet. The improvements in our approach with respect to [12] can be summarized in terms of the following new features:

1. provision of MIMO capabilities through the use of antenna arrays at both the transmitter and receiver sides;
2. addressing the estimation of the downlink channel affected by noise;
3. testing the system with a 5G NR fully compliant simulator with a clustered delay line channel model.

The remainder of this paper is organized as follows: Section 2 introduces CSI-RS, which are reference signals used for downlink channel sounding. Section 3 provides a description of the New Radio link simulator software developed for all our experiments in order to outline the simulation environment. Section 4 presents the NR-CsiNet DL model, describing its internal structure and the data-processing steps required. The simulation results are presented in Section 5. Section 6 concludes the article with some observations and suggestions for potential future research directions.

## 2. Channel State Information Reference Signals

One of the key design principles of NR is to avoid, as far as possible, “always-on” signals [21,33]. In release 8 of the LTE standard, channel sounding for downlink transmissions is performed by means of device measurements on cell-specific reference signals (CRS) [34], which are transmitted over the whole transmission bandwidth in each subframe. From release 10 on [35], these reference signals are complemented by another type of reference signal called CSI-RS, which, in contrast to the first, is not expected to be continuously transmitted. The main reason for this upgrade lays in the necessity to support spatial multiplexing with more than four layers; however, as [21] points out, CSI-RS introduction has opened the way to further technological extensions, such as coordinated multi-point (CoMP) [36] operations and interference estimation.

In 3GPP specifications [37], the channel quality indicator (CQI), rank indicator (RI), precoding matrix indicator (PMI), and several other quantities, are jointly referred to as channel state information (CSI). The precoding matrix indicator is reported by the user equipment (UE) to the base station (BS); it represents an indication of what the user considers to be a suitable precoding matrix to be applied on downlink transmissions. In particular, the PMI is no more than a set of indexes or a single index pointing to a specific entry in the precoding codebook, which contains a list of all the possible precoding matrices W that the device can select and report to the network. There exists at least one codebook for each permitted combination of $N_{T}$ (number of antenna ports) and $N_{L}$ (number of layers) associated with the configured CSI-RS.

It is important to stress that precoding codebooks play a role only in the context of PMI reporting and do not impose any restriction in the choice of the precoding matrix to be applied by the network [21]. In some cases, it is convenient for the network to select the precoding matrix suggested by the device, but, when, for example, the network is in possession of additional information, the precoding computation may lead to a different choice.

As [21] reports, the typical use case of multi-antenna precoding is multi-user MIMO (MU-MIMO) [38,39,40]. In this case, the main purposes are to direct the energy towards the device and simultaneously limit the interference towards the other users scheduled on the same time-frequency resource. In this kind of scenario, it is clear that the network needs more detailed information about the channel experienced by different UEs so that it can take into account the PMI reported by all simultaneously scheduled devices when selecting the precoding matrix [21].

The NR standard defines two types of CSI reporting, each characterized by a different size and structure of the codebook [37]:

- Type I CSI codebooks: designed for single user-MIMO (SU-MIMO) scenarios, where a single user is scheduled within a certain time-frequency resource, transmitting on a potentially large number of layers in parallel;
- Type II CSI codebooks: designed for MU-MIMO scenarios, where many users are scheduled within the same time-frequency resource, each transmitting on a limited number of layers in parallel (two at most).

For fast time-varying wireless channels, the adoption of delayed CSI at the transmitter can help reduce the feedback frequency of the channel estimates [41].

## 3. New Radio Link-Level Simulator

New Radio link simulator software was used for all the experiments conducted and described in this paper. The simulator was developed in Telecom Italia laboratories with an engine implemented in MATLAB^®^. The most critical blocks in terms of execution speed were, instead, implemented in C language and linked to the MATLAB engine via MEX. The purpose was to model a radio interface which was fully compliant with 3GPP specification and to evaluate the link-level performances of 5G-based point-to-point communications. The simulator is a link-level simulator and targets scenarios with a single next-generation base station or gNodeB and a single UE for a SU-MIMO scenario where the UE can deploy an antenna array.

**Figure 1:**
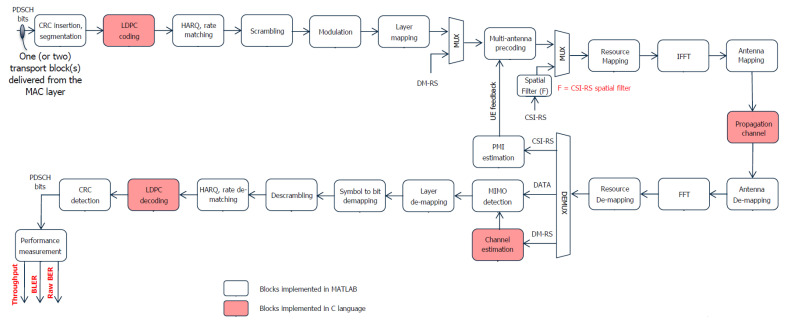
New Radio link-level simulator scheme.

The various blocks of the simulator provide a bit-level model of the 5G NR physical layer, as described in the 38 series of the 3GPP specifications [37,42,43,44]. The physical downlink shared channel (PDSCH) transmission chain can be represented as the block scheme of Figure 1, which also reflects the 5G NR simulator structure. The red blocks are those which exploit the C language to speed-up the computations.

### 3.1. Transmission Chain

A detailed description of the main blocks is provided as follows.

***Transport Block generator*** 
Data are generated as a sequence of bits. Depending on the transport size (A) and the modulation and coding scheme (MCS), in particular, on the code rate (R), the LPDC base graph selection is performed:
  - if A≤292, or if A≤3824 and R≤0.67, or if R≤0.25, LDPC base graph 2 is used;
  - otherwise, LDPC base graph 1 is used.
  Input bits are then segmented into different blocks; the CRC parity bits are calculated on and attached to each block. Depending on the LPDC base graph, the maximum dimension of blocks can change. The lowest order information bit is mapped to the most significant bit of the transport block (i.e., parity bits are found at the end of the block).
**LDPC encoder**  LPDC coding is applied to the bit sequence for a given code block. *N* encoded bits are obtained as output, where $N=66\;Z_{c}$ for LDPC base graph 1 and $N=50\;Z_{c}$ for LDPC base graph 2 ($Z_{c}$ value can be found in subclause 5.2.2 of TR 38.212 [42]).
**Rate-matching block**  A rate-matching block is required to adapt the size of the transport block after LDPC coding to the size of the allocated resources that can be used for PDSCH transmission. PDSCH coded bits must be rate-matched around the resource elements occupied by the CSI-RS and other reference signals, otherwise the coding rate may exceed the unit value (i.e., the REs in the slot available for PDSCH are not sufficient to transmit the information bits). In practice, it is expected that the gNB scales down the MCS in the slots where the CSI-RS transmission causes an LDPC coding rate higher than about 0.95. This approach is also modelled in the link simulator. The throughput calculation must consider this effect, re-scaling the formula accordingly.
**Scrambling operation**  Before modulation, the codewords (up to two) are scrambled, as in subclause 7.3.1 of [42], resulting in a block of scrambled bits.
**QAM modulation**  The block of scrambled bits for each codeword are QAM-modulated using one of the modulation schemes (selected by the MCS) from table 7.3.1.2-1 [42], resulting in a block of complex-valued modulation symbols.
**Layer and subcarrier mapping**  The modulated symbols are mapped to one or more layers (if multi-layer transmission is enabled), by splitting the codewords (one or two) among them. Then, the symbols are inserted into the actual resource elements of the physical resource block, through a predefined look-up table, based on the 3GPP resource allocation procedure.
**MIMO processing**  The precoding matrix is applied to the PDSCH channel, i.e., sub-carriers containing data. If PMI reporting is enabled, the precoding matrix $F_{j}$ is selected by the transmitter at the gNB as the *j*-th element of the codebook, where *j* is the PMI index reported by the UE. The digital beam-forming scheme implemented in the simulator is wideband; that is, the same beam-forming matrix $F_{j}$ is applied to all the PRBs. If, instead, the channel estimate is given through feedback by the UE, (e.g., SRS transmission in uplink), downlink beam-forming schemes based on the channel matrix can be applied exploiting the downlink/uplink channel reciprocity.
**CSI-RS mapping**  The transmission of CSI-RS signals in the link-level simulator is modelled in a realistic way. The CSI-RS signals are generated and mapped in the time frequency-grid and configuration parameters set the number of ports and the transmission period, denoted as *CSI-RS period*, which can take the set of values defined in the 3GPP TS 38.331 [45] specifications. The CSI-RS signals are transmitted once every [4, 5, 8, 10, 16, 20, 32, 40, 64, 80, 160, 320, 640] slots.
**OFDM modulator**  The OFDM modulation is performed through the IFFT Operation. Before the IFFT, an IFFT shift must be applied to properly translate the spectrum into the positive frequency domain. The IFFT must be applied on an OFDM symbol-basis. The channel bandwidth and sub-carrier spacing (SCS) determine the size of the IFFT and the number of samples for each OFDM symbol. At this point a cyclic prefix is added at the beginning of each OFDM symbol, through a look-up table, with a duration selected between “normal” or “extended”. The “normal” option is selected for the simulations according to the used sub-carrier spacing of 30 kHz. This value is compatible with the maximum delay of the channel that falls into the selected CP length.
**Oversampling and filtering**  An oversampling step increases the number of samples for each symbol by a selected factor and the sampling frequency is increased accordingly. A raised cosine filter is used as the transmitting filter.
**Receiver side**  At the receiver side, a finite impulse response filter is applied to remove the undesired frequencies and a down-sampler stage re-samples the signal to the normal frequency, by the same factor as the up-sampler. To account for the delay due to the signal transmission and FIR filter, at each iteration, the signal at the receiver side is buffered and processed on a block basis, where the block length in time is equal to the slot duration. The receiver must then reverse all the signal-processing applied by the transmitter with suitable blocks, such as an OFDM signal demodulator, a sub-carrier demapper, and so on. The MIMO processing consists of the application of a combining matrix over the received symbols to exploit the antenna array at the receiver if more than one antenna is configured. The combining matrix can be used for various purposes, i.e., improving the SINR or for separating the different layers if more than one data stream is transmitted using spatial multiplexing. The LDPC decoder applies the offset min-sum (OMS) decoding algorithm. The performance measurements are applied on the CRC detection for the BLER, while the raw BER is computed on the number of actual erroneous bits before the LDPC decoding. The approximate peak throughput of the simulated NR configuration can be calculated by means of the formula provided in the TS 38.306 3GPP [46] specification:
  (1) $$\mathrm{data}\;\mathrm{rate}\;\left[\mathrm{Mbps}\right]=10^{-6}\cdot \sum_{j=1}^{J}\left(v_{L}^{\left(j\right)}\cdot Q_{m}^{\left(j\right)}\cdot f^{\left(j\right)}\cdot R_{max}\cdot \frac{N_{PRB}^{BW(j),\mu }\cdot 12}{T_{s}^{\mu }}\cdot \left(1-OH^{\left(j\right)}\right)\right)$$
  where:
  - *J* is the number of aggregated component carriers in a band or band combination.
  - $R_{max}=948/1024$ is the maximum LDPC code rate.
  - $v_{L}^{\left(j\right)}$ is the maximum number of layers.
  - $Q_{m}^{\left(j\right)}$ is the maximum modulation order
  - $f^{\left(j\right)}$ is the scaling factor (it can take the values 1, 0.8, 0.75 and 0.4) and is signalled per band or per band combination).
  - μ is the numerology.
  - $T_{s}^{\mu }$ is the average OFDM symbol duration in a subframe for numerology μ and normal CP, i.e., $T_{s}^{\mu }=\frac{10^{-3}}{14\cdot 2^{\mu }}$.
  - $N_{PRB}^{BW(j),\mu }$ is the maximum RB allocation in maximum UE supported bandwidth BW(j) with numerology μ.
  - $OH^{\left(j\right)}$ is the overhead (0.14 for DL FR1-0.18 for DL FR2-0.08 for UL FR1-0.10 for UL FR2).

### 3.2. Channel Model Generation

The key element of this simulation framework is clearly the fading channel block: for an NR-compliant link simulator, the 3GPP has two defined models for NR link and system evaluation purposes [22,47]:

- Tapped delay line (TDL): for this channel model, the correlation between different antennas is defined statically by a correlation matrix. The TDL model is based on the description of the impulse response of the channel;
- Clustered delay line (CDL): with this channel model the direction of the signal in the space is modelled. The model is based on the description of the main departure and arrival directions of the signal in the space and the number of clusters corresponds to the number of channel reflections.

The CDL channel model is adopted for all the simulations presented in this article due to its importance for practical applications. In particular, we select the CDL-B profile. This CDL profile is specific for non-line-of-sight (NLOS) transmissions and can be used for sub 6 GHz frequency, where line-of-sight (LOS) is not mandatory. A characterising property is that the first path in time is also the strongest one.

The channel between the user and the BS depends on the environment geometry. In our scenario, the user is placed at a distance of 100 m from the BS, with a height of 1.5 m, while the BS has a height of 10 m. The UE direction could be defined as DOMINANT_PATH (the same direction of the main azimuth of arrival (AoA) direction), RANDOMLY (uniformly generated around $\left[-180^{{}^{\circ}},+180^{{}^{\circ}}\right]$) and ARBITRARY (statically selecting a UE direction). The delay spread can be selected among the set (‘VERY SHORT’, ‘SHORT’, ‘NOMINAL’, ‘LONG’, ‘VERY_LONG’). NOMINAL is fixed for our set of simulations, which corresponds to 100 ns. The coherence time of the channel depends on the Doppler spread and on the user speed, which is fixed at 3 km/h (i.e., a walking pedestrian). The CDL profile channel requires the generation of $N_{c}$ clusters. Each cluster *n* will be assigned a different delay (depending on the delay spread times), a set of angular orientations, including AoA, azimuth of departure (AoD), zenith of departure (ZoD) and zenith of arrival (ZoA), and a power gain $P_{n}$. The azimuth angle spread of departure and arrival (ASD and ASA, respectively) and the zenith angle spread of departure and arrival (ZSD and ZSA, respectively), characterize the entire set of clusters. These values are used for the generation of the *M* rays that form each cluster. The direction of a certain ray is given by the angular orientation of its own cluster, the angular spreads and a different coefficient for each ray. The angle for azimuth is ϕ, while the angle for zenith is θ. At both the gNB and UE side, three levels of antenna correlation are possible: LOW, MEDIUM and HIGH. The level of correlation is inversely related to the angle spread (i.e., larger angle spreads correspond to lower correlation and vice versa). The CDL model for cluster *n*, transmitter antenna element *u* and receiver antenna element *s* is, thus, a combination of *M* NLOS rays and a LOS ray when the UE is in a LOS condition:

(2) $$\begin{array}{cc} H_{u,s,n}^{\mathrm{NLOS}}= & \sqrt{\frac{P_{n}}{M}}\sum_{m=1}^{M}\;{\left[\begin{array}{c} F_{rx,u,\theta }\left(\theta_{n,m,\mathrm{ZoA}},\phi_{n,m,\mathrm{AoA}}\right)\\ F_{rx,u,\phi }\left(\theta_{n,m,\mathrm{ZoA}},\phi_{n,m,\mathrm{AoA}}\right) \end{array}\right]}^{⊺}\left[\begin{array}{cc} e^{\left(j\Phi_{n,m}^{\theta \theta }\right)} & \sqrt{\kappa_{n,m}^{-1}}e^{\left(j\Phi_{n,m}^{\theta \phi }\right)}\\ \sqrt{\kappa_{n,m}^{-1}}e^{\left(j\Phi_{n,m}^{\phi \theta }\right)} & e^{\left(j\Phi_{n,m}^{\phi \phi }\right)} \end{array}\right]\\ \; & \left[\begin{array}{c} F_{tx,s,\theta }\left(\theta_{n,m,\mathrm{ZoD}},\phi_{n,m,\mathrm{AoD}}\right)\\ F_{tx,s,\phi }\left(\theta_{n,m,\mathrm{ZoD}},\phi_{n,m,\mathrm{AoD}}\right) \end{array}\right]\exp\left(\frac{j2\pi {\hat{r}}_{rx,n,m}^{T}\cdot {\bar{d}}_{rx,u}}{\lambda_{0}}\right)\exp\left(\frac{j2\pi {\hat{r}}_{tx,n,m}^{T}\cdot {\bar{d}}_{tx,s}}{\lambda_{0}}\right)\exp\left(\frac{j2\pi {\hat{r}}_{rx,n,m}^{T}\cdot \bar{v}}{\lambda_{0}}\right) \end{array}$$

(3) $$\begin{array}{c} H_{u,S}^{\mathrm{LOS}}={\left[\begin{array}{c} F_{rx,u,\theta }\left(\theta_{LOS,\mathrm{ZoA}},\phi_{\mathrm{LOS},\mathrm{AoA}\;}\right)\\ F_{rx,u,\phi }\left(\theta_{LOS,\mathrm{ZoA}},\phi_{\mathrm{LOS},\mathrm{AoA}}\right) \end{array}\right]}^{⊤}\left[\begin{array}{cc} 1 & 0\\ 0 & -1 \end{array}\right]\left[\begin{array}{c} F_{tx,s,\theta }\left(\theta_{\mathrm{LOS},\mathrm{ZoD}},\phi_{\mathrm{LOS},\mathrm{AoD}}\right)\\ F_{tx,s,\phi }\left(\theta_{\mathrm{LOS},\mathrm{ZoD}},\phi_{\mathrm{LOS},\mathrm{AoD}}\right) \end{array}\right]\exp\left(\frac{-j2\pi {\bar{d}}_{3D}}{\lambda_{0}}\right)\\ \exp\left(\frac{j2\pi {\hat{r}}_{rx,LOS}^{T}\cdot {\bar{d}}_{rx,u}}{\lambda_{0}}\right)\exp\left(\frac{j2\pi {\hat{r}}_{tx,LOS}^{T}\cdot {\bar{d}}_{tx,s}}{\lambda_{0}}\right)\exp\left(\frac{j2\pi {\hat{r}}_{rx,LOS}^{T}\cdot \bar{v}}{\lambda_{0}}\right) \end{array}$$

where *F* is the field pattern in the vertical or horizontal plane; ${\hat{r}}_{tx,n,m}$ and ${\hat{r}}_{rx,n,m}$ are the ray *m* direction vectors in spherical units, heading from the BS and reaching the UE, respectively; $\bar{d}$ is the location vector of the antenna elements; ${\bar{d}}_{3D}$ is the distance between the BS and the UE; $\hat{v}$ is the user speed; and $\lambda_{0}$ is the wavelength corresponding to the carrier frequency. Moreover, $\left\{\Phi_{n,m}^{\theta \theta }\Phi_{n,m}^{\theta \phi }\Phi_{n,m}^{\phi \theta }\Phi_{n,m}^{\phi \phi }\right\}$ are the random initial phases for each ray *m* of each cluster *n* and for four different polarization combinations (θθ,θϕ,ϕθ,ϕϕ), with distribution $∼U(-180^{{}^{\circ}},+180^{{}^{\circ}})$, and $\kappa_{n,m}$ is the cross-polarization power ratio (XPR) for each ray *m* of each cluster *n* (7.5-21 in [22]).

The channel generation implementation in the link-level simulator is obtained by several blocks. All the blocks launch the same mex-file with a different configuration:

- *cdlModelInit*: during the configuration of the link simulator parameters, the channel is initialized and the memory is allocated on the basis of parameter selection (e.g., the allocated memory grows with the number of antenna elements). The *mexLock()* function is used to reserve memory to the mex application.
- *cdlModelRegen*: during both the initialization and running phase, the main AoA/AoD of the clusters can be modified to analyze multiple MIMO configurations in a single simulation.
- *cdlModelRun*: the CDL is generated each slot and is applied to the input data. The time-to-frequency conversion of the channel is applied to report the generated channel matrix as a function of the frequency.
- *cdlModelDelete*: at the end of the simulation, the memory reserved to the mex application is deallocated using the *mexUnlock()* function.

The simulator can then operate in two different modes: the frequency domain or the time domain. In the time domain mode, the channel is applied on the oversampled OFDM signal after the IFFT. In the frequency domain mode, the channel is applied in the frequency domain on the OFDM signal before the IFFT. In this case, the OFDM modulator and demodulator are by-passed, as are all the blocks between them, allowing a significant reduction in the computational load. However, the frequency domain mode is applicable only in scenarios characterized by low mobility (i.e., when the channel is nearly constant within the OFDM symbol duration) and when the maximum delay of the channel is lower than the cyclic prefix length. In the simulations presented in the following, the time domain mode is selected and an impulse response is generated after a certain time interval, defined as an integer multiple of the slot length, and then interpolated in the time domain. For the frequency domain, an impulse response would be generated for each OFDM symbol.

## 4. A Deep Learning-Based CSI Feedback for New Radio

To obtain the optimal precoder for MIMO communications, the channel knowledge is required by the transmitter (i.e., the BS). In the frequency division duplexing (FDD) mode, the uplink and downlink transmissions occur in different frequency bands, so there is no channel reciprocity. Thus, the UE must estimate the downlink channel and send this estimation back to the BS. This procedure is the channel state information feedback. The drawback of this system is the unbearable overhead resulting from the higher number of antenna elements, both in transmission and reception, as highlighted in [12]. The only way to adopt the FDD mode for MIMO transmission is to significantly reduce the feedback overhead—machine learning represents one viable solution to address the problem. In [12], the author designed a DL-based CSI sensing and recovery mechanism, named CsiNet. The main difference from previous DL-based investigations was consideration of the recovery stage. The CsiNet model is based on two main modules:

- Encoder: the encoder generates compressed codewords by learning a proper mapping from the estimated channel matrices (dimensionality reduction). It is implemented by the UE.
- Decoder: the decoder tries to recover the original CSI from the codewords generated by the encoder, learning the inverse mapping. It is implemented at the BS sides, whereas the codewords are received from the UE through a feedback channel.

In this paper, we extend the CsiNet model for CSI information reduction and recovery in a 5G context, producing a deep convolutional neural network (CNN), named NR-CsiNet. The additional features are intended to widen the applicability of the model to a more practical range of use cases:

- We sought to make the model compatible with the typical 5G NR MIMO communications, in contrast to the single receiving antenna limitation of [12]. Practically, this introduces a third dimension to the channel matrices due to the different channel coefficients for each receiving antenna.
- We also considered the downlink channel estimation, whereas this additional step was beyond the scope of [12]. This means that, instead of feeding our NR-CsiNet with perfect channel matrices, we provide as input, noisy matrices, estimated through the CSI-RS.

The CNN is built and trained via Tensorflow’s Keras API [48]. To make this DL-based system work with the 5G NR simulator, the traditional feedback reporting blocks are rearranged. A new block imports the pre-trained encoder and uses it to elaborate an encoded version of the channel matrix, previously estimated from the CSI-RS. Then, at the transmitter side, we insert a specular decoding block which imports the pre-trained decoder model and uses the reconstructed channel matrix at its output to determine the optimal precoding vector.

### 4.1. System Model

While the authors of [12] adopted a multiple-input single-output (MISO) system, i.e., a transmitter deploying an antenna array of *N* antennas with only one antenna at the receiver side, here, we consider a 5G NR-compliant MIMO system, with *M* receiving antennas. For the *c*-th sub-carrier at the *m*-th receiving antenna, the received signal can be expressed as

(4) $$y_{m,c}=h_{m,c}^{H}v_{c}x_{c}+z_{m,c}$$

where:

- $h_{m,c}$ is the complex channel vector relative to the *c*-th sub-carrier and to the *m*-th receiving antennas of size N×1;
- $v_{c}$ is the complex precoding vector of size N×1;
- $x_{c}$ is the complex data symbol transmitted on the *c*-th sub-carrier;
- $z_{m,c}$ denotes the additive noise on the *c*-th sub-carrier relative to the *m*-th receiving antenna.

The precoding vector $v=\{v_{c}:c=1,\ldots ,\tilde{C}\}$, with $\tilde{C}$ the number of sub-carriers of the OFDM system, is computed by the BS once it receives the CSI feedback information $H_{c}=\left[h_{1},\ldots ,h_{M}\right]\in C^{N\times M}$. The amount of coefficients the UE must feedback is then $N\times M\times \tilde{C}$, which can easily become too demanding from a complexity point of view, since resource elements must be dedicated to the channel reporting, instead of data packets (i.e., a capacity drop). The *M* $y_{m,c}$ symbols received per sub-band are then combined at the receiver side and the final estimated symbol ${\hat{y}}_{c}$ on the *c*-th sub-carrier is obtained as follows:

(5) $${\hat{y}}_{c}=u_{c}^{H}y_{c}$$

where $u_{c}$ is an M×1 combiner and $y_{c}={\left[y_{1,c},\ldots ,y_{M,c}\right]}^{T}$ is an M×1 vector containing all the received symbols on the *c*-th sub-carrier.

### 4.2. The Channel Estimation Task

When considering massive MIMO systems, the channel estimation task is fundamental and many studies have addressed the issue [19,20] since beam-forming techniques strongly depend on the outcome of this phase. In [12], the main focus is the compression task, i.e, the model in [12] takes as input a perfect channel matrix and learns efficient encoding and decoding algorithms to compress and decompress it with minimum error. Our model, instead, aims to deal also with the challenging downlink channel estimation task. This means that the compression and decompression process is not performed on the ideal channel matrix generated by the CDL channel module, but on a noisy estimation of it, coming from the CSI-RS compensation. The model is expected to learn efficient encoding and decoding algorithms that, besides compressing and decompressing the input matrices, must be able to remove the noisy contributions and reconstruct the clean channel matrices with minimum error. It should be noted that, to simplify the experiments, we decided to consider only the noise affecting the downlink transmission to avoid simulating the uplink channel.

To include this channel estimation task, we need to define two different instances of the channel matrix H:

- an ideal channel matrix $H^{id}$, generated by the CDL module of the software simulator. It represents the desired output of the NR-CsiNet model;
- a noisy version of the perfect channel matrix $H^{\mathrm{CSI}-\mathrm{RS}}$, estimated from the CSI-RS pilots by means of a simple interpolation. It represents the input of the NR-CsiNet model.

To obtain a first raw estimation of the complete downlink complex channel matrix for each transmitting/receiving antenna pair, we performed a cubic radial basis function (RBF) interpolation on the real and the imaginary parts separately, using a MATLAB predefined library.

**Figure 2:**
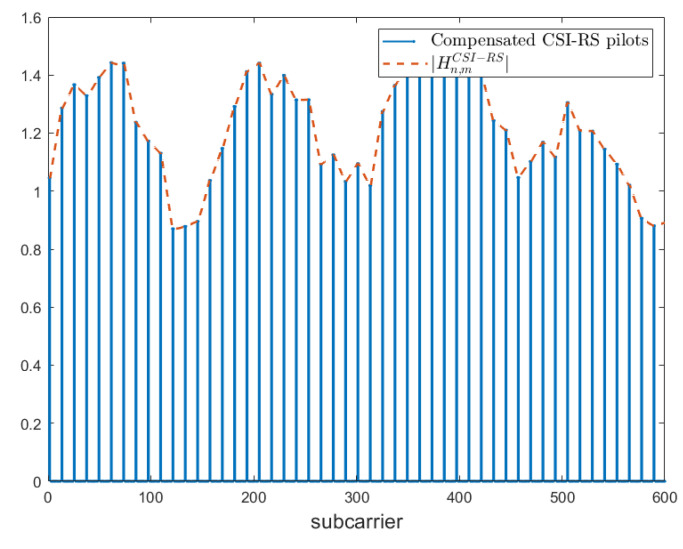
Module of interpolated channel matrix $|H_{n,m}^{\mathrm{CSI}-\mathrm{RS}}|$.

An example of the result obtained is shown in Figure 2, where *n* and *m* are the transmitting and receiving antenna indexes, respectively. The estimated $H^{\mathrm{CSI}-\mathrm{RS}}$ matrices are collected by simulating different conditions of the channel; practically, we select a couple of different SNR values, 10 dB and 20 dB, and train a different NR-CsiNet model for each of these values. Then, we test the behavior of these models at different levels of channel quality; to be precise, simulations are performed with SNR values varying between −5 dB and 35 dB, with a 5 dB granularity. To simplify the notation, in the following subsections, we refer to the two channel instances $H^{id}$ and $H^{\mathrm{CSI}-\mathrm{RS}}$ as a single matrix H, meaning that all the processing steps described have to be performed on both.

### 4.3. DFT-Based Preprocessing of CSI Information

**Figure 3:**
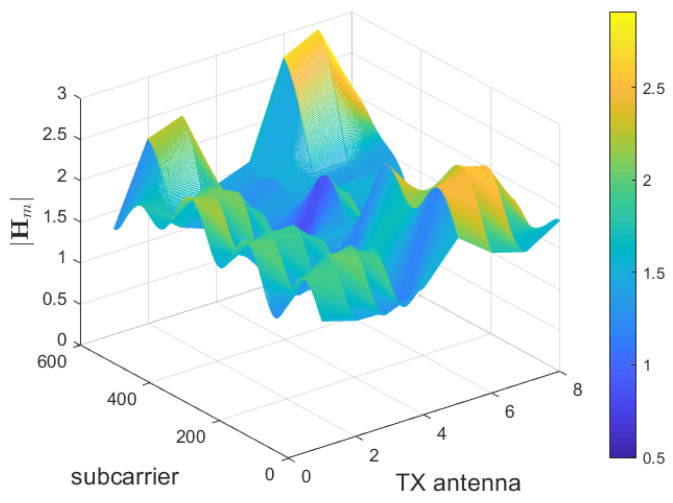
Module of channel matrix $|H_{m}|$ [32].

The first processing that is applied to the estimated channel matrix in the feedback task is the 2D discrete Fourier transform (DFT), with the goal of sparsifying the coefficients of H in the angular-delay domain, as proposed in [12]. To deal with more antennas at the receiver, this operation is iterated *M* times, thus producing a different transformed spatial-frequency channel matrix ${\tilde{H}}_{m}$ for each receiving antenna:

(6) $${\tilde{H}}_{m}=F_{d}H_{m}F_{a}^{H}$$

where $F_{d}$ and $F_{a}$ are $\tilde{C}\times \tilde{C}$ and N×N DFT matrices, respectively. $H_{m}$ has size $\tilde{C}\times N$ and *m* is the index of the receiving antenna. Figure 3 shows an instance of $H_{m}$ with N=8 and $\tilde{C}=600$.

**Figure 4:**
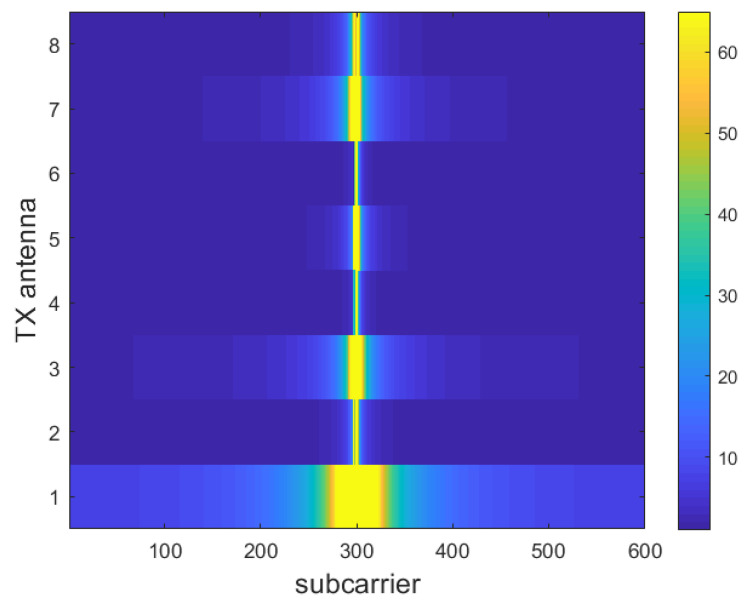
Module of DFT-transformed channel matrix $|{\tilde{H}}_{m}|$ [32].

As the inter-arrival time between rays only spans a certain range, only a few elements of the matrix ${\tilde{H}}_{m}$ contain significant information, while the vast majority of the matrix elements are made up of coefficients close to zero; thus, DFT processing is necessary. It is obvious that the remaining columns of ${\tilde{H}}_{m}$ can be eliminated and that only the core $C<\tilde{C}$ columns are significant (Figure 4).

A truncated version of ${\tilde{H}}_{m}$ is then obtained as a first step to decrease the feedback overhead, denoted as ${\tilde{H}}_{m}^{t}$ and having size C×N.

Successively, we obtain the spatial-frequency domain version of the truncated matrix in the angular-delay domain ${\tilde{H}}_{m}^{t}$, through an an inverse DFT (IDFT). This matrix, denoted $H_{m}^{t}$, eases the compression and reconstruction task of the CNN, since, in the spatial-frequency domain, the “image” representation of the matrix is characterized by a smoother profile (Figure 3), whereas, in the angular-delay domain, it is recognizable as a composite of crisp and sharp lines (Figure 4). This last step is not considered in the DFT processing addressed in [12]; we anticipate that it will improve the overall system behaviour.

Lastly, the matrix $H_{m}^{t}$ is normalised to assume values only inside the interval [0,1]. This can help the training of the CNN by reducing the effect of outliers.

In a specular way, at the output of the decoder implemented at the BS, a rescaling operation, followed by a DFT and an inverse DFT operation, must be performed.

### 4.4. Multiple Receiving Antennas

While the authors of paper [12] limit their DL-based solution to single receiving antenna scenarios (M=1), our purpose is to make the model applicable also to MIMO contexts. With this in mind, the downlink channel information relative to each distinct receiving antenna must, in some way, be organized at the input of the CNN. We decided to maintain only two input channels, one for the real and one for the imaginary parts of the matrices $H_{m}^{\mathrm{CSI}-\mathrm{RS},t}$; this means that the matrices relative to distinct receiving antennas are conveyed through the CNN on the same input channels.

The reasons behind this approach are several:

- we do not need to train a different CNN model for each distinct receiving antenna;
- the number of parameters learned by the CNN is not affected by the number of receiving antennas *M*. This means that in massive MIMO scenarios, where M≫1, the complexity of the model does not increase with *M* and, thus, the risk of running into overfitting phenomena remains limited;
- if the different receiving antennas were spatially correlated, this correlation could be exploited during the learning process and, probably, smaller training datasets would be sufficient to obtain effective and generalized models.

### 4.5. NR-CsiNet Encoder

**Figure 5:**
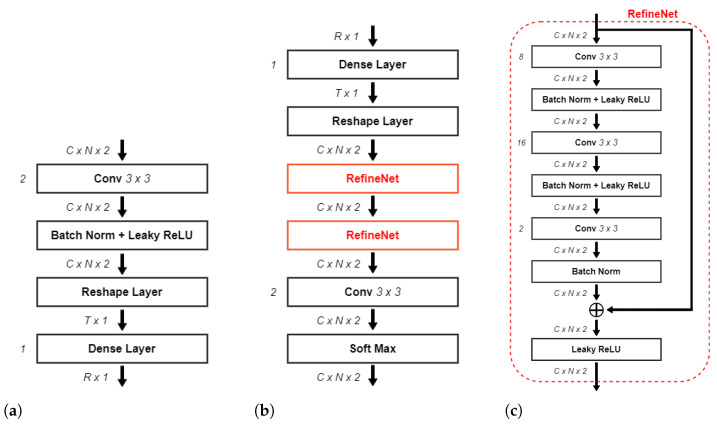
NR-CsiNet representation. (**a**) Block scheme of the NR-CsiNet encoder. (**b**) Block scheme of the NR-CsiNet decoder. (**c**) Block scheme of the RefineNet unit.

The structure of the encoder of the NR-CsiNet (Figure 5a) is based on the encoder designed for the CsiNet proposed in [12]. It consists of a deep convolutional neural network, where the input has size C×N×2, corresponding to the real and imaginary parts of the truncated matrices $H_{m}^{\mathrm{CSI}-\mathrm{RS},t}$. The first layer of the CNN is a convolutional layer adopting a filter of size 3×3 that generates 2 feature maps. A reshape process vectorizes the two feature maps into a vector of size T×1, where T=2·C·N. The last block is a fully connected layer which outputs the actual codeword s, one for each receiving antenna, as a vector of size *R*. The overall compressing ratio is $K=\frac{R}{\tilde{C}\cdot N}$.

### 4.6. NR-CsiNet Decoder

As for the encoder, the NR-CsiNet decoder (Figure 5b) utilizes the implementation scheme of the CsiNet decoder presented in [12]. The first layer is a fully connected layer which receives as input the codeword s produced by the encoder at the UE side, sent to the BS. The result of this layer is a coarse reconstruction of the real and imaginary part of the channel matrix $H_{m}^{id,t}$ in the form of a vector of size T×1. A reshape layer transforms the vector back to the two matrices corresponding to the real and imaginary part of the channel, i.e., two matrices of size C×N each. In order to improve these initial estimates, a particular set of blocks, called the RefiNet Unit (Figure 5c), is inserted in the decoder. Each RefineNet unit is composed of three convolutional layers using a kernel of size 3×3. The first and second layers generate 8 and 16 feature maps, respectively, while the third outputs the final reconstruction of the $H_{m}^{id,t}$ matrices (in the form of two feature maps). The feature maps produced by the three layers of a RefineNet unit are forced by a zero-padding technique to have the same size as the input channel matrix (C×N). Moreover, each layer is followed by a rectified linear unit (ReLU) [49,50] and a batch normalisation layer [50,51]. Before the last leaky ReLU computation, the input of the RefiNet is added to the output of the last CNN (after proper batch normalisation). This step is necessary since, as reported in the literature [52], the sequence of many CNN layers may lead to a vanishing gradient problem. To identify a suitable trade-off between the complexity and reconstruction capabilities, the authors of [12] investigated the impact of the number of RefiNet units, determining that two RefineNet units were sufficient. In our work, we investigated the effect of adopting different numbers of RefiNet units and observed that exploiting more than two RefiNet units did not significantly impact the reconstruction quality, though at the expense of greater complexity. An additional convolutional layer follows the RefiNet Units and, finally, a softmax layer [50,53] scales the reconstructed coefficients into the [0,1] range.

### 4.7. Training Process

Using the same notation adopted in [12], the set of trainable parameters is denoted as $\Theta =\{\Theta_{en},\Theta_{de}\}$. The truncated input and output of the NR-CsiNet model for the *i*-th patch are denoted as $H_{i}^{\mathrm{CSI}-\mathrm{RS},t}$ and ${\hat{H}}_{i}^{t}=f\left(H_{i}^{\mathrm{CSI}-\mathrm{RS},t};\Theta \right)=f_{de}\left(f_{en}\left(H_{i}^{\mathrm{CSI}-\mathrm{RS},t};\Theta_{en}\right);\Theta_{de}\right)$, respectively.

The loss function is defined as

(7) $$L\left(\Theta \right)=\frac{1}{S}\sum_{i=1}^{S}||f\left(H_{i}^{\mathrm{CSI}-\mathrm{RS},t};\Theta \right)-H_{i}^{id,t}{\left|\right|}_{2}^{2}$$

where *S* denotes the number of training samples and ${\left||.|\right|}_{2}$ is the Euclidean norm operator.

## 5. Simulation Results

In this section, we report the main results collected from the NR-CsiNet model and described in the previous sections. The assessment of the performance is carried out through simulation trials exploiting the NR link-level simulator presented in Section 3 by substituting the blocks for CSI feedback definition and reporting with our DL-based models. The transmitter precoding matrix is obtained by a single value decomposition (SVD) operation on the recovered channel matrix, which is generated from the compressed feedback sent back by the UE. This means selecting the eigenvector that corresponds to the higher eigenvalue as the precoding vector for the subsequent downlink transmission. Taking advantage of the spatial diversity given by the *M* receiving antennas, a maximal ratio combiner (MRC) is used at the receiving side.

Table 1 reports the main parameters of interest concerning the simulator settings:

More details on some of the main parameters are provided below:

**Time slot**  Time duration of the transport block of data transmission, corresponding to 14 OFDM symbols.
**Modulation and coding scheme (MCS)**  Configuration parameter of the system to determine the coding rate and the order and type of modulation to be adopted for the transmission.
**Transport block size (TBS)**  Value corresponding to the number of bits transmitted in one slot.
**NFFT**  Smaller power of two greater than the number of sub-carriers used by the system to be used by FFT and IFFT operations.
**Number of sub-carriers for data transmission**  $\tilde{C}$ Computed as the difference between the NFFT value and the number of sub-carriers composing the band guards. In our system $\tilde{C}=512-106-106$.
**Delay spread model**  Parameter that determines the delay profile, affecting the scaling factor for the time of arrival of each cluster signal, leading to a shorter or longer delay spread.

It should be noted that the adopted bandwidth is smaller than the one commonly expected for a 5G NR network. Behind this choice are considerations of computational complexity since a larger bandwidth would lead to a significant increase in simulation and training time.

Table 2 shows the hyperparameters adopted for the training and testing process of our model.

The DFT-compression factor $K_{\mathrm{DFT}}$, which should not be confused with the final compression factor *K* introduced in Section 4.5, is defined as:

(8) $$K_{\mathrm{DFT}}=\frac{\tilde{C}}{C}\;$$

where $\tilde{C}$ and *C* are the same parameters defined in Section 4.

The transmitting antenna system consists of a 2×2 planar array formed of dual-polarized antenna components, while the receiver is equipped with a single dual-polarized antenna; thus, we have N=8 transmitting antennas and M=2 receiving antennas. Since there are a relatively small number of antennas, it is obvious that this is not a massive MIMO scenario. This decision was forced by considerations solely related to computational complexity because adding more antennas will increase simulation times proportionately. Larger values of *N* and/or *M* should be envisioned for future research.

**Figure 6:**
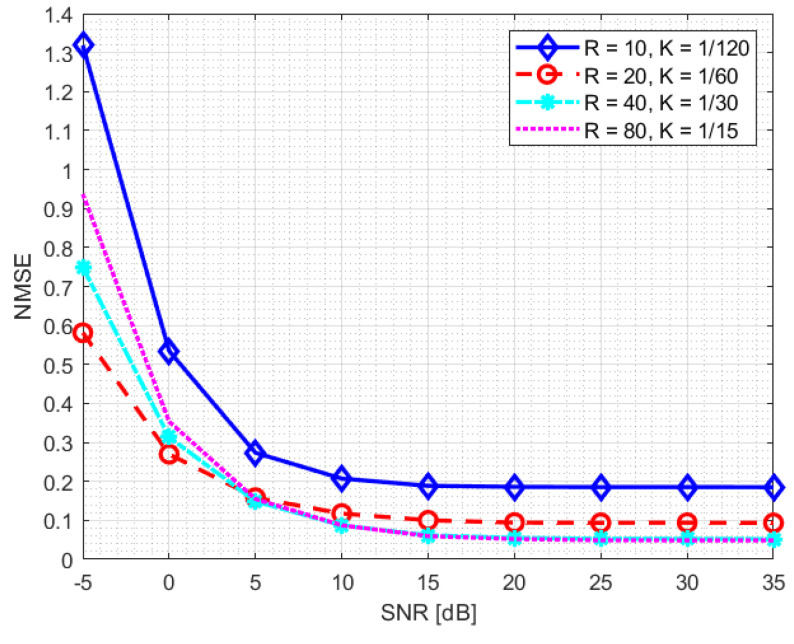
NMSE statistics for NR-CsiNet model trained at SNR = 10 dB.

**Figure 7:**
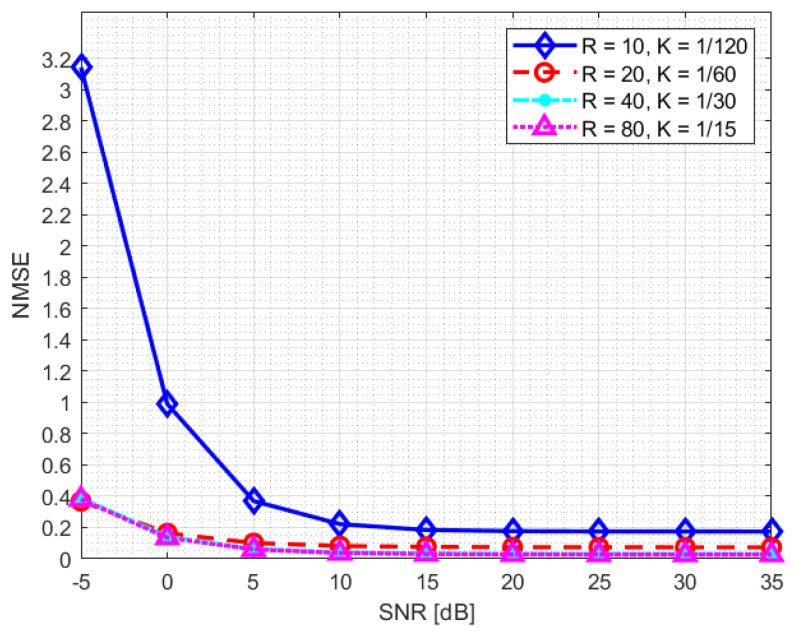
NMSE statistics for NR-CsiNet model trained at SNR = 20 dB.

In our system, the DFT-compression factor is fixed at $K_{\mathrm{DFT}}=3$. The training process of the neural network is performed for two different SNR levels: 10 dB and 20 dB. Figure 6 and Figure 7 highlight how the normalised mean square error (NMSE) of the channel reconstruction for the NR-CsiNet model does not depend on the training data SNR level, except for the lowest compression value *K* (K=1/120).

**Figure 8:**
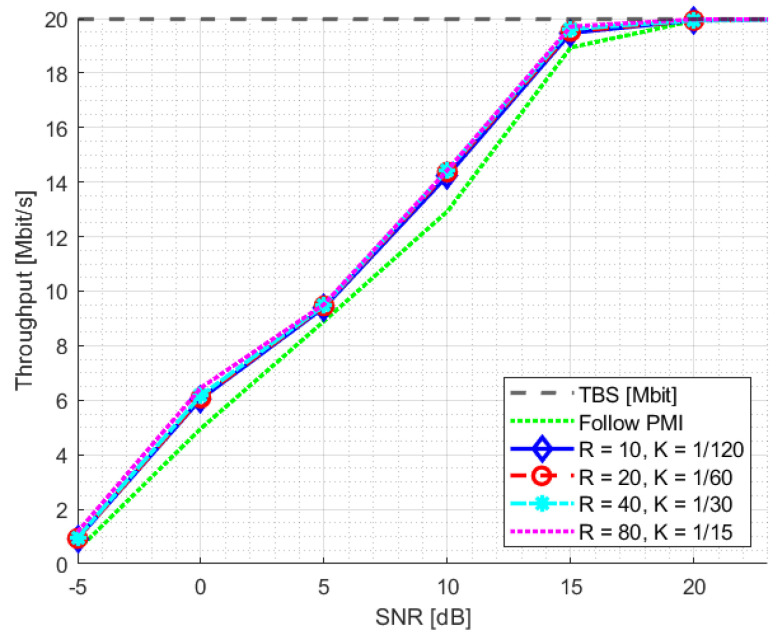
Throughput statistics for NR-CsiNet model trained at SNR = 10 dB.

**Figure 9:**
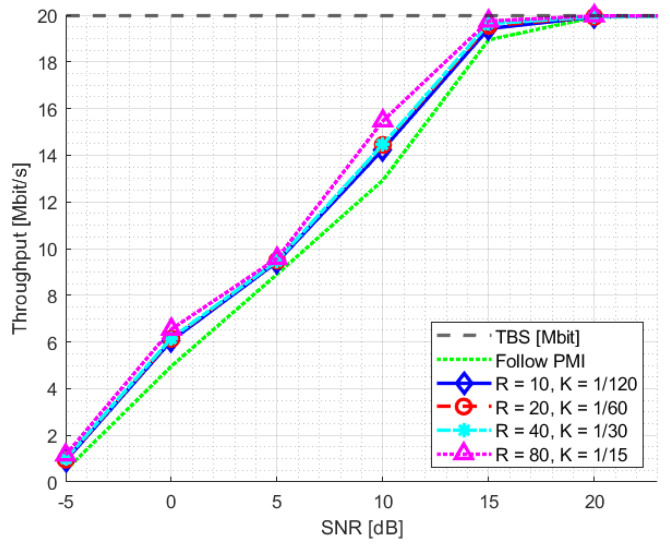
Throughput statistics for NR-CsiNet model trained at SNR = 20 dB [32].

**Figure 10:**
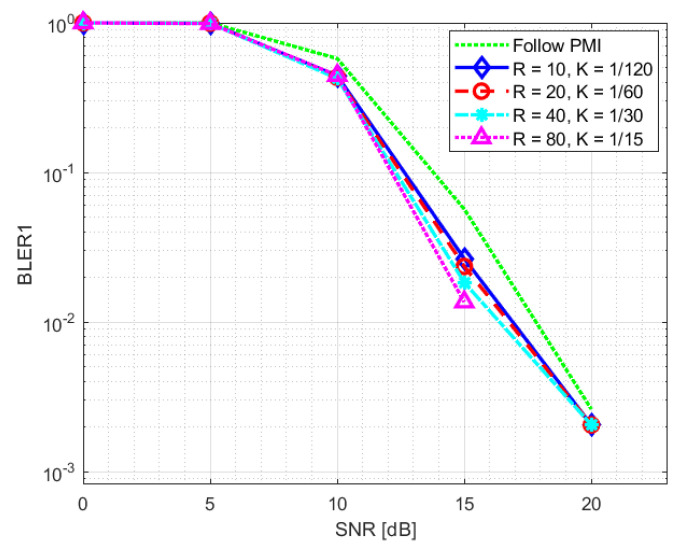
BLER1 statistics for NR-CsiNet model trained at SNR = 10 dB.

**Figure 11:**
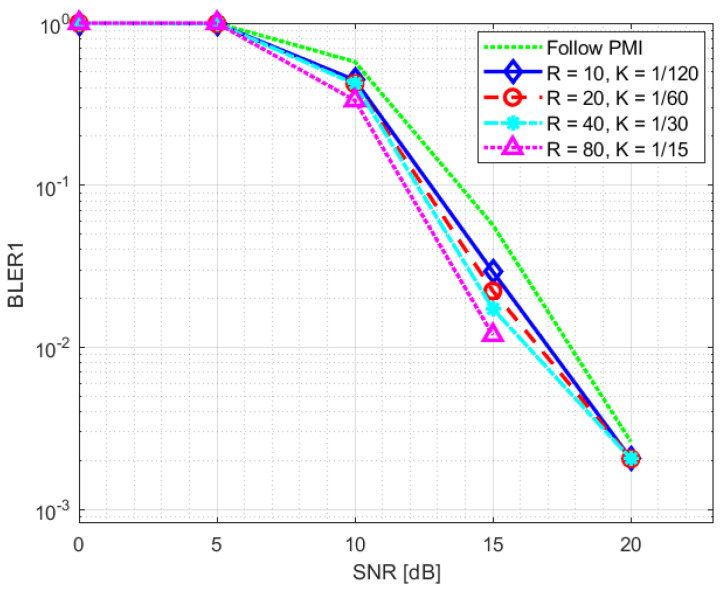
BLER1 statistics for NR-CsiNet model trained at SNR = 20 dB [32].

The PDSCH throughput results for SNR training values of 10 dB and 20 dB are reported in Figure 8 and Figure 9, respectively. The relevant SNR range is [−5,23] dB. The results for the second evaluation metric, BLER1, i.e., BLER at the first transmission attempt, are reported in Figure 10 and Figure 11 for the [0,23] dB SNR range. The performance of the NR-CsiNet is compared with the PMI reporting technique, referred to here as follow PMI. This procedure requires channel measurements by the UE from CSI-RS, in addition to which a PMI is determined [37]. The PMI is an index that the UE sends back to the BS to suggest the preferable precoding vector corresponding to that index from a predefined precoding matrix.

It is possible to infer from the graphs that, even when dealing with noisy channel estimates rather than ideal channel matrices, our DL-based technique performs better than the follow PMI algorithm in 8×2 cases. The plots specifically illustrate that over 20 dB of SNR, both the NR-CsiNet and follow PMI throughput curves achieve the transport block size (TBS) maximum limit; however at lower SNR, all the variations of our DL solution perform better. It is important to note that, in all of our experiments, we used the same channel model configuration. The simulation results demonstrated that, despite being intended for multipath contexts, this type of channel model typically presents a dominant path, which implicitly defines a preferential direction for signal transmission. It is obvious that the follow PMI is expected to function well when taking into account such a channel model. In fact, among a range of potential beams, the PMI index merely determines which one is most precisely pointed to the receiver. Our hypothesis is that our DL-based technique has the best chance of outperforming the follow PMI algorithm in “richer” channel realizations when the multipath is not dominated by a single LOS cluster.

The complexity of DL-based solutions is one of the main shortcomings in their implementation at the user equipment side, characterized by limited power and hardware resources. In Table 3, we report the number of parameters and FLOPs required for the NR-CsiNet encoder, which are greatly dependent on the choice of the factor *K*. For the CNN layer, the number of learnable parameters are computed as $par_{CNN}=\#\;filters\cdot \left(filter\;width\cdot filter\;height\cdot input\;feature\;maps+1\right)$ while the FLOPs are $FLOP_{CNN}=2\cdot \#\;filters\cdot filter\;width\cdot filter\;height\cdot Output\;size$; for the FC layer, the parameters are $par_{FC}=\left(Input\;size+1\right)\cdot Output\;size$, while the FLOPs are $FLOP_{FCL}=2\cdot Input\;size\cdot Output\;size$.

The FLOP amount is of the same order of magnitude for both the CNN and the FC, while the FC requires a significantly higher number of parameters since it needs a weight for each input-output node link (plus the bias). The advanced hardware designed specially for neural network operations can significantly help the deployment of such technology. For the follow PMI, the parameters required are almost negligible, since the DFT codebook can be generated whenever required. Nonetheless, the complexity from a FLOPs perspective is almost zero at the base station; however, at the user side it can be quite significant, since it requires a full search over all the DFT beam-forming codewords in each frequency position to maximize the capacity. Thus, the number of FLOPs can be estimated as the multiplication between two complex matrices of size M×N and $N\times C_{s}$: $FLOP_{followPMI}=8MC_{s}NL$; where $C_{s}$ is the codebook size and *L* is the number of frequency bins where the PMI is computed. In our tests, a different PMI is required for each PRB; thus, L=25 and we use as the codebook a DFT beamspace with an oversampling factor $O_{F}=4$; therefore, $C_{s}=O_{F}N=32$. The final number of FLOPs with follow PMI is equal to $FLOP_{followPMI}=102.400$. The use of a system such as NR-CsiNet instead of the simpler follow PMI demands higher complexity; however, unlike follow PMI, it can allow the BS to apply advanced precoding techniques that exploit complete CSI, which is not achievable through simplified codebooks (e.g., in the case of users in NLOS and/or who could interfere with each other). We aim to investigate NR-CsiNet capabilities for more sophisticated MIMO scenarios in future studies.

## 6. Conclusions

The CSI feedback for FDD transmission is an open problem and DL-based techniques have made a new tool available to attempt to solve it. The channel state information availability is of crucial importance for MIMO communications and for 5G New Radio. In this paper, we investigated the potential of a neural network technique to serve as an alternative to the traditional feedback reporting block. From the work of the authors reported in [12], we obtained a NR-CsiNet model, able to deal with SU-MIMO systems and providing channel estimation capabilities. Simulations were performed by exploiting a 5G NR-compliant physical layer simulator with a CDL channel model, which enables modelling of the complex environment envisioned by the 5G network. The results showed that our DL-based feedback system can achieve performances comparable to those obtained with a traditional PMI-based reporting technique and clearly outperformed it in some cases. The framework and the data collected in this study provide a solid basis for more in-depth tests of the capabilities of DL for CSI feedback in 5G NR, enabling investigation of a larger number of scenarios and different configurations, such as larger MIMO systems (i.e., massive MIMO), and exploitation of the channel matrix to support spatial division multiplexing solutions, that are currently difficult to obtain in FDD mode.

Moreover, our objective was to highlight useful aspects of research on this topic, which is of significant interest considering the fundamental role that MIMO systems play in network performance improvement.

## Figures and Tables

**Table 1:** Simulation parameters for NR-CsiNet training.

NR Simulator Parameters	
System bandwidth	5 MHz
Subcarrier spacing	15 kHz
Time slot	1 ms
Carrier frequency	3.64 GHz
iMCS	21
(Modulation, coding rate)	(64-QAM, 0.694)
TBS	19,968 bit
NFFT	512
$\tilde{C}$	300
Transmission layers	1
Codewords	1
CSI-RS Tx period	4 slots
CSI reporting type	Type I
Channel model	CDL-B
Delay spread model	Nominal

**Table 2:** NR-CsiNet model parameters [32].

NR-CsiNet Parameters	
Training set	1000 sim of 100 slots
Validation set	100 sim of 100 slots
Testing set	100 sim of 100 slots
Learning rate	0.001
Loss function	MSE
Optimizer	Adam
Epochs	300
RefineNet units	2
$K_{\mathrm{DFT}}$	3

**Table 3:** Complexity of the encoder in terms of learnable parameters and FLOPs.

K	Parameters	FLOPs
1/120	5.381	179.200
1/60	10.725	243.200
1/30	21.411	371.200
1/15	42.785	627.200

## Data Availability

The data that support the findings of this study are available from TIM S.p.A. Restrictions apply to the availability of these data, which were used under licence for this study. Data are available from the corresponding author, D.G.R., with the permission of TIM S.p.A., upon reasonable request.
